# How Often, where and by whom are Adverse Experiences Recorded in Clinical Records of Service-Users Under the Care of an Early Intervention in Psychosis (EIP) Service?

**DOI:** 10.1007/s10597-025-01529-x

**Published:** 2025-10-25

**Authors:** Hazel Davison, Matt Sopp, Alison Bennetts

**Affiliations:** 1 Department of Psychology, University of Southampton, Southampton, UK; 2 Hampshire and Isle of Wight Healthcare NHS Foundation Trust, Hampshire, UK

**Keywords:** Adverse experiences, Psychosis, EIP, Clinical records, Audit

## Abstract

Research indicates high prevalence rates of adverse experiences in people experiencing first-episode psychosis. Despite recommendations that mental health staff should routinely ask about adverse experiences, documentation indicates these are not being recorded in service-users’ clinical records across different mental health settings. This study audited 48 service-user records in a UK early intervention in psychosis (EIP) community mental health service to examine how often, where, and by whom adverse experiences were recorded. Searching for 22 adverse experience terms, 64.6% of clinical records documented at least one adverse experience, with 80.6% reporting more than one. The profession that most often recorded adverse experiences in service-users’ clinical records was psychiatrists. While EIP services may document adverse experiences more frequently than other community mental health settings, recorded rates remain lower than expected based on previous research prevalence rates. Further studies should consider adverse experiences recording across UK EIP services to ensure service-users with adverse experiences receive appropriate support.

## Introduction

Adverse experiences are known risk factors in the onset and maintenance of a range of mental health conditions (Hogg et al., 2022; McKay et al., 2021, 2022). In addition, childhood adversity is related to more severe psychiatric outcomes (Longden et al., 2016). Childhood abuse increases the likelihood of being acutely suicidal on admission to inpatient psychiatric care with increased duration in hospital (Read, 1998) and reduces the age of onset of psychosis with increased chance of relapse (Goff et al., 1991). Prevalence rates of trauma are significantly higher in patients with severe mental illness compared to the general population and documentation of this trauma in service-users’ medical records is exceptionally low (Mauritz et al., 2013). The UK government has outlined guidance on the key principles and implementation of trauma-informed care (Office for Health Improvement & Disparities, 2022). However, implementation of trauma-informed care has been disjointed and there is a need for a more coordinated provision with increased funding (Emsley et al., 2022).

Childhood adverse experiences are markedly more prevalent in people with psychosis compared to other mental health conditions (Bebbington et al., 2004) with a meta-analysis finding significant associations between five out of the six childhood adverse experiences studied and an increased risk of psychosis (Varese et al., 2012). Morgan et al. (2014) found exposure to childhood adverse experiences creates a vulnerability for psychotic experiences, and Croft et al. (2019) found, in a sample of over 4,000 UK individuals, that 83.8% of 18-year-olds with psychotic symptoms had experienced some form of trauma compared to 62.6% with no psychotic symptoms. A review paper (Stanton et al., 2020) found significant evidence that individuals who experience adverse childhood experiences have an increased risk of psychosis and are at an increased risk of their psychosis being worse in severity. Multiple exposure to childhood adversity further increases the risk of developing psychosis in adulthood (Pastore et al., 2022), with research indicating three or more childhood adverse experiences is associated with a 4.7 fold increase in the odds of experiencing psychosis (Croft et al., 2019) and an average increase in the odds of experiencing psychosis of 1.45 for every additional adverse life experience (Morgan et al., 2014).

Considering service-users with first episode psychosis (FEP), Trauelsen et al. (2015) found that in a sample of 101 FEP patients, 89% reported experiencing one or more childhood adverse experiences compared to 37% in a non-clinical control group. Furthermore, 52% reported 3 or more adversities compared to 7% in the control group, and that for every extra adversity the risk of psychosis increased by 2.5 times. These trends are supported by Aas et al. (2016) who found significantly more childhood adverse experiences were reported in a sample of 96 FEP service-users compared to healthy controls from the same catchment area, and that childhood trauma led to greater severity of clinical symptoms at both baseline and one year follow up.

The high prevalence rate of adverse experiences experienced by service-users with severe mental illness suggests the need for these experiences to be routinely asked about by mental health professionals. Read et al. (2005) recommended that all mental health services should establish guidelines for how and when to ask about adverse experiences and that service-users with psychosis should be routinely asked about trauma as they are unlikely to spontaneously disclose childhood adversity. The Department of Health (2008) guidelines recommend that all mental health staff routinely ask service-users about adverse experiences. Further research since the publication of these guidelines has also recommended routine assessment of co-occurring trauma in youth presenting with psychotic symptoms (Stanton et al., 2020). By routinely asking about adverse childhood experiences, clinicians can develop comprehensive formulations and treatment plans when working with service-users with psychosis (Varese et al., 2012).

However, research indicates that adverse experiences are not being routinely asked about. A literature review (Hepworth & McGowan, 2013) found that childhood sexual abuse is not routinely asked about by mental health professionals in acute health settings. Although in critique, this study used literature searches from 2008–2009; at a similar time to when the Department of Health (2008) published their guidance around asking about adverse experiences. More recently Sellick et al. (2020) found that in a sample of 213 mental health professionals, over half did not routinely ask about sexual abuse in service-users presenting with psychotic symptoms. However, this study looked only at Australian mental health professionals so cannot be generalised to UK healthcare services. A systematic review (Read et al., 2018a, b) looking at whether adult mental health services identify childhood abuse and neglect found that only 28% of abuse and neglect cases identified by researchers are being reported in the service-user’s record, with only 22% of service-users reporting that they were asked by clinicians about child abuse. A review of nearly 12 million UK primary care records found a substantial underreporting of maltreatment and abuse (Chandon et al., 2020). Interestingly, a literature review also found that even when service-users are asked about childhood adverse experiences the inclusion of this in formulations is between 12%−57%, reducing to 12%−44% for inclusion in treatment plans and further reducing to just 8%−23% for onward referrals to appropriate therapy (Read et al., 2018a, b).

Longden et al. (2016) found that in the community mental health team records of 215 service users, of which 23.1% had a primary diagnosis of a psychotic disorder, 56.2% had at least one form of adverse childhood experience recorded. Neill and Read (2022) looked at the frequency of asking about, documentation of, and responding to disclosures of adverse experiences in UK Community Mental Health Teams (CMHTs) in the records of 400 service-users, of which 83.8% had a psychotic disorder. They found that 87% of core assessments contained no documentation about adverse experience and that service-users with a psychotic diagnosis were less likely to have adverse experiences documented. This research suggests a substantial under recording of adverse experiences in service-user electronic records when compared to prevalence rates of adverse experiences noted in the previous literature for service-users with psychosis.

As the research to date was carried out in CMHTs in which a number of psychiatric presentations are served, there is a need to look at how adverse experiences are being recorded in psychosis-specific community mental health services. Reeder et al. (2017) looked at the case notes of 296 patients in a UK EIP service and found that 60% of individuals had experienced some sort of trauma, but that 19% of records had insufficiently documented whether childhood trauma had occurred and 26% had no evidence of childhood trauma. However, the study was completed seven years ago and only looked at childhood trauma. More recently, Wood et al. (2023) looked at trauma-informed care in EIP services across five countries including the UK and found that 52% of the 124 EIP healthcare professionals asked shared concerns about current trauma related policies, and only a minority of services indicated that trauma was being formally assessed. Mountjoy et al. (2024) asked 11 clinical psychologists from UK EIP services using a vignette-based scenario about the extent to which they ask about adversity and trauma during an assessment and found around half were routinely asking about this. However, the study used a hypothetical scenario, with a small sample and did not directly look at service-users’ electronic records. As such, there is a need for further up-to-date research into the extent to which adverse lifetime experiences are being recorded in the electronic records of service-users under the care of UK EIP services.

The aim of the present study was to investigate how frequently childhood and adulthood adverse experiences were recorded in the clinical records of service-users in one UK EIP service. The primary research question was:

- To what extent are adverse experiences recorded in the electronic clinical records of service-users under the care of an EIP service?

The secondary research questions were:

- When adverse experiences are recorded in a service-user’s electronic clinical record, what is the profession of the clinician who recorded the adverse experience?
- When adverse experiences are recorded in a service-user’s electronic clinical record, in which documents are they being recorded?

## Method

### Design

The study was a quantitative audit of secondary data available in service-users’ electronic NHS medical records. The primary variable was the prevalence rate of adverse experiences occurring in either childhood or adulthood recorded in the electronic medical records of service-users under the care of an EIP community mental health service. This was considered against the prevalence rates reported in the literature (Croft et al., 2019; Trauelsen et al., 2015). The secondary variables were the profession of the clinician who recorded the adverse experience in the service-user’s electronic record and where in the service-user’s electronic record the adverse experience was recorded. The electronic records of service-users who had opted out of participating in NHS research and audits via the national opt-out scheme were excluded from the audit.

### Participants

The audit looked at the electronic records of 49 service-users on the accepted caseload of an EIP community mental health service. The EIP service supported individuals aged 14–65 experiencing their first episode of psychosis, with service-users typically discharged after three years unless earlier discharge was deemed appropriate due to no longer requiring care or disengagement. Service-users’ data were excluded if they had been accepted by the service for less than three months or were due to be discharged within six months of the commencement of data collection. The rationale for this was to allow time for service-users to be assessed upon acceptance into the service and to reduce the chance of them being discharged during data collection. A total of 98 service-users met criteria for inclusion having been accepted into the service more than three months prior and with an expected discharge date of more than six months after the commencement of data collection. The sample of 49 service-users were selected using systematic sampling with a fixed starting point by listing service-user electronic records by date of acceptance to the EIP caseload and starting with the oldest record. The sampling interval was determined by creating a sampling frame of the records which met inclusion criteria (98), determining the sampling size (half the possible records due to service limitations so 49) and then calculating the sampling interval by dividing the total number of records by the desired sample size (sampling interval = 2). Starting from the chosen starting point of the oldest record every 2^nd^ record was selected until the desired sample size was reached. The age range of the sample was 19–61 years which falls within, and is consistent with, the target age of individuals accessing EIP services (14–65 years). The gender split of the sample was 45% female to 55% male.

### Procedure

The study was granted ethical approval by the University of Southampton (ERGO number: 90292). Permission to access service-users’ electronic records as part of this audit was given by the EIP service. Data was collected by searching the electronic NHS medical records of 49 service-users currently on the accepted caseload of an EIP Service. The data was collected between January 2024 and March 2024. Service-users’ individual electronic clinical notes and documents (referrals, formulations, assessments and discharge letters) were searched for adverse experience related terms. These terms were decided on by reviewing the Adverse Childhood Experience (ACE) Questionnaire for Adults (Felitti et al., 1998), the Trauma and Life Events (TALE) Checklist (Carr et al., 2018) and previous relevant research (Longden et al., 2016; Neill & Read, 2022; Varese et al., 2012). The search terms used were: “trauma”, “psychological trauma”, “adverse childhood experience”, “traumatic event”, “sexual abuse”, “physical abuse”, “emotional abuse”, “psychological abuse”, “non-specific childhood trauma”, “parental loss during childhood”, “parental death”, “parental separation”, “military deployment to a warzone”, “maltreatment”, “victimisation”, “discrimination”, “family mental health issues”, “neglect”, “bullying”, “death of a sibling”, “domestic violence” and “parent in prison”. Due to the time constraints of the study, only the recording of these exact terms was collected, and service-user electronic records were not read in their entirety.

Adverse experiences documented in each service-users’ electronic clinical notes or documentation were recorded on a password protected spreadsheet, alongside the profession of the clinician (psychiatrist, care-coordinator, psychologist, occupational therapist, support worker, mental health nurse, physical health specialist, CBT therapist, administration team, carer support worker, peer support worker, management or social worker) and where the adverse experience was first recorded (referral letter, formulation, assessment letter, discharge letter or clinical note). Each service-user was allocated a participant number to ensure they were pseudo-anonymised upon completion of the data collection. The researcher used an additional password protected spreadsheet to store service-users’ first name and surname initial alongside their allocated participant number to keep track of which records had been searched; this was deleted upon completion of data collection.

### Data Analysis

Data was inputted and analysed using IBM SPSS version 29 (IBM Corp., 2022). The first time an adverse experience was documented in a service-user’s electronic record was recorded alongside the profession of the clinician and where it was recorded. This was repeated for each specific adverse experience. Percentages were calculated for the number of service-users electronic records which included the mention of any adverse experience. Descriptive statistics were calculated for the total number of adverse experiences for each service-user, which profession was most likely to record an adverse experience, where that adverse experience was recorded and how often each search term was recorded.

## Results

A total of 48 electronic records were searched due to one service-user being discharged from the EIP caseload before completion of data collection. From the 48 electronic records searched, 64.6% (31 service-users) had at least one of the adverse experience search terms recorded either in their clinical notes or documentation (referral letter, assessment, formulation or discharge letter).

**Fig. 1:**
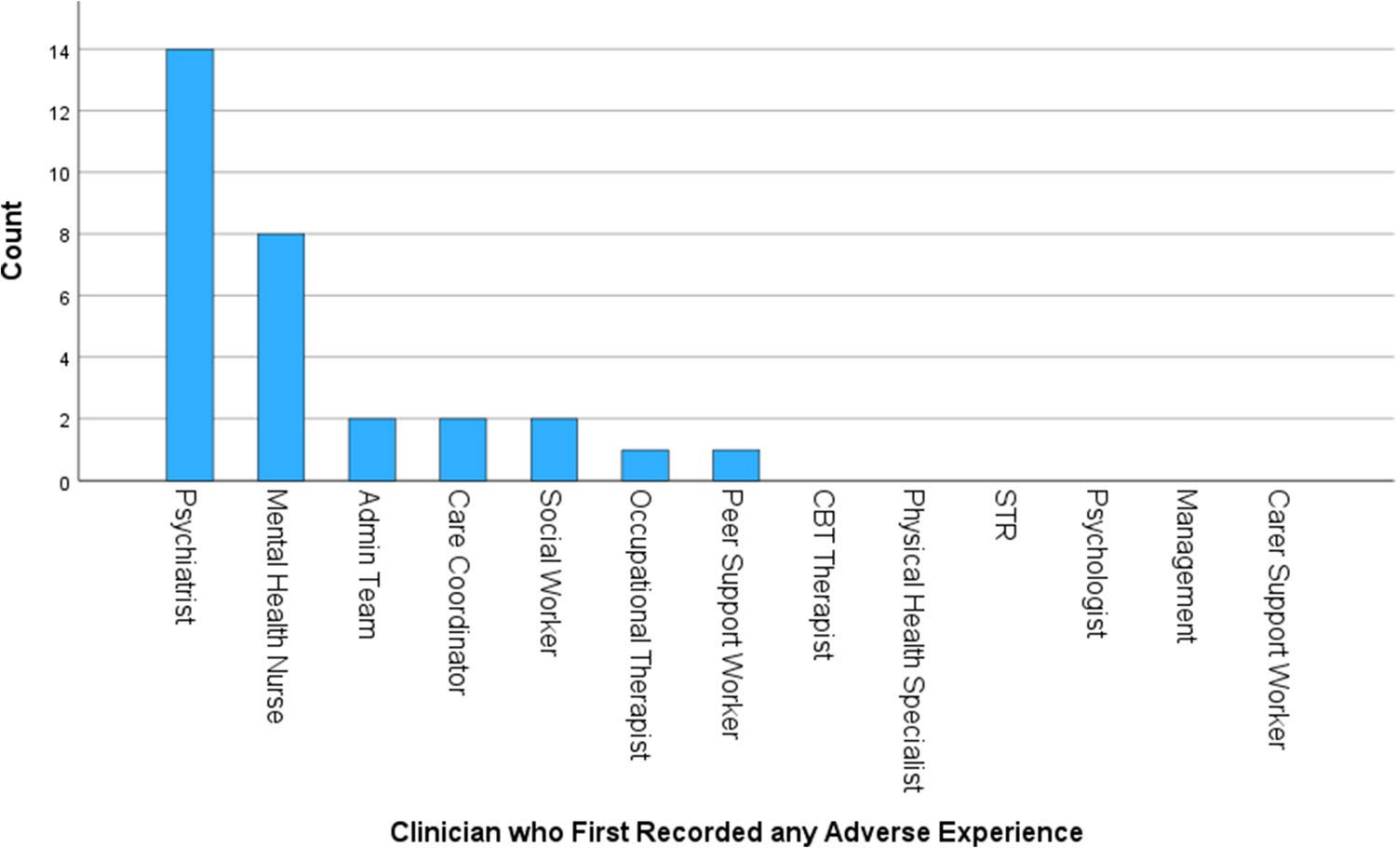
The profession of the clinician who first recorded any type of adverse experience in the service-user’s record

Figure 1 shows the profession of the clinician who first recorded any of the adverse experience search terms in a service-user’s record. The results show that this was most commonly completed by psychiatrists (45% of the time, *n* = 14); 26% of the time it was recorded by a mental health nurse (*n* = 8); 6% of the time it was recorded by either the administration team (*n* = 2), a care-coordinator (*n* = 2) or a social worker (*n* = 2), and 3% of the time it was recorded by an occupational therapist (*n* = 1) or a peer support worker (*n* = 1). On no occasions was the adverse experience first recorded by a psychologist, CBT therapist, physical health specialist, support time recovery (STR) worker, carer support worker or the management team. In one case of an adverse experience being recorded the profession of the clinician was unclear.

**Fig. 2:**
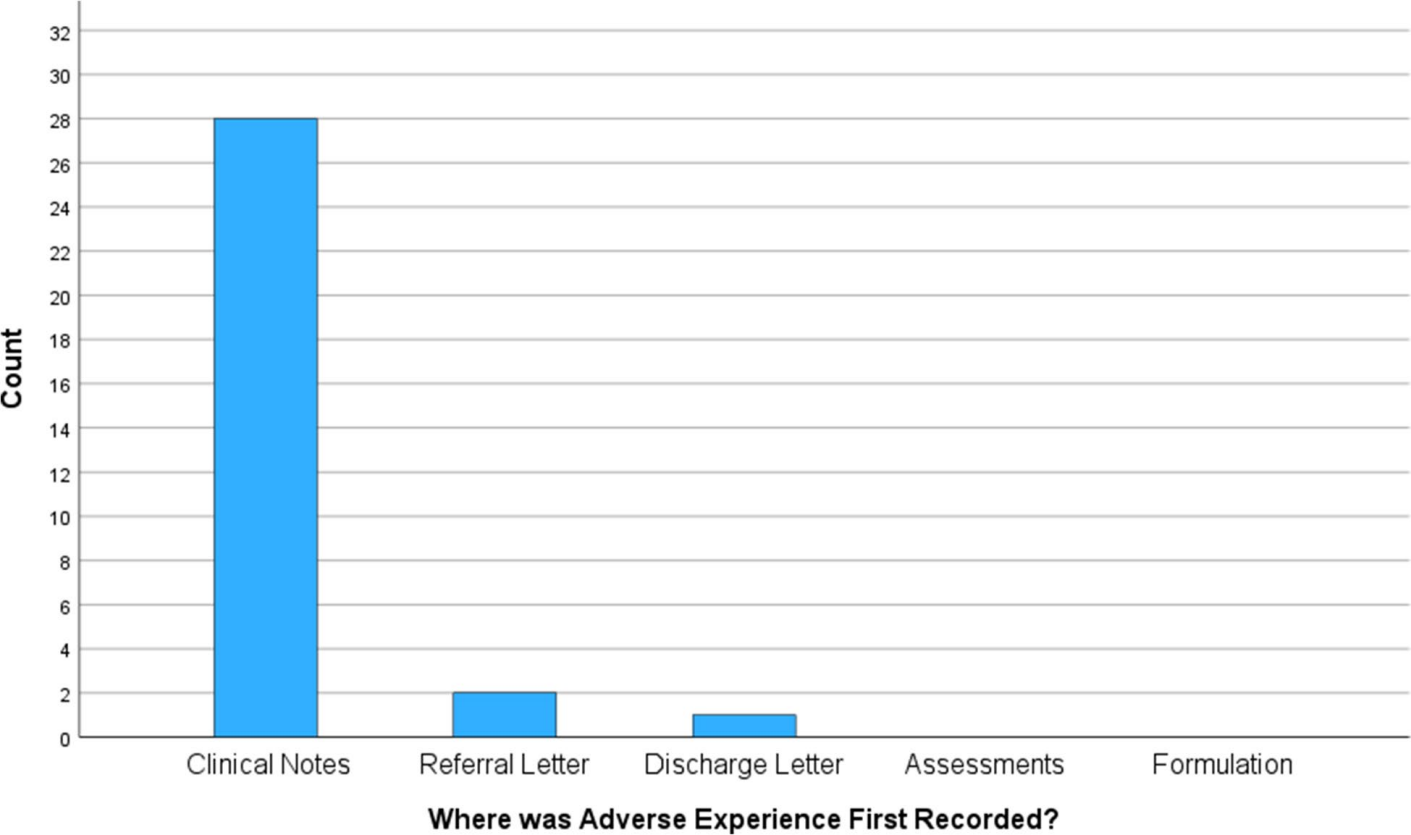
Bar chart of where in a service-user’s record adverse experiences were first recorded

Figure 2 shows where the first recording of an adverse experience was recorded. The results show that adverse experiences were most commonly recorded in a service-users’ clinical notes (90% of the time) on two occasions the adversity was recorded in the referral letter (6%) and once it was recorded in a discharge letter (3%). On no occasions was the adverse experience first recorded in an assessment letter or a formulation document, which had been separately uploaded to the service-users’ electronic records.

Regarding the individual types of adverse experiences recorded in service-users’ electronic records, the number of adverse experiences ranged from one to nine, with 80.6% (25 records) containing more than one type of adverse experience and 41.9% (13 records) containing three or more adverse experiences (Table 1). The modal number of adverse experiences recorded was two.

**Table 1:** The frequency of service-users with multiple adverse experiences recorded in their clinical records

Number of adverse experiences recorded	Frequency of service-users	Percentage of service-users
1	6	19.3%
2	12	38.7%
3	6	19.3%
4	3	9.7%
5	0	0
6	3	9.7%
7	0	0
8	0	0
9	1	3.2%

Further exploratory analysis of the individual adverse experience search terms is displayed in Table 2, including the number of times each search term was recorded, the profession who most often recorded each adverse experience, and where each adverse experience was most often recorded. The most common adverse experience search term recorded was trauma (80.6%). For 11 of the 23 search terms (47.8%), the adverse experience was most commonly first recorded by a psychiatrist and for 17 of the 23 search terms (73.9%), the service-user’s clinical notes was the first place where the adverse experience was recorded.

**Table 2:** The frequency of each adverse experience search term, who it was recorded by and where

Adverse experience search term	Number of times recorded	Percentage of 31 records where at least one adverse experience was recorded	Recorded most often by which profession	Frequency recorded by that profession	Recorded most often where	Frequency of times it was recorded in most common place
Trauma	25	80.6%	Psychiatrist	11	Clinical note	23
Psychological Trauma	1	3.2%	Psychiatrist	1	Clinical note	1
Adverse Experience	1	3.2%	Care-coordinator	1	Clinical note	1
Adverse Childhood Experience	2	6.5%	Psychiatrist/Care-coordinator	1 1	Clinical note	2
Traumatic Event	5	16.1%	Mental Health Nurse	3	Clinical note	4
Sexual Abuse	8	25.8%	Psychiatrist	4	Clinical note	7
Physical Abuse	8	25.8%	Psychiatrist/Social Worker	2 2	Clinical note	8
Emotional Abuse	5	16.1%	Mental Health Nurse	2	Clinical note	5
Psychological Abuse	3	9.7%	Mental Health Nurse	2	Clinical note	2
Non-Specific Childhood Trauma	3	9.7%	Psychiatrist	2	Clinical note	3
Parental Loss During Childhood	1	3.2%	Psychiatrist	1	Assessment document	1
Parental Death	2	6.5%	Psychiatrist/Care-coordinator	1 1	Clinical note	2
Parental Separation	1	3.2%	Mental Health Nurse	1	Clinical note	1
Military Deployment to a Warzone	1	3.2%	Mental Health Nurse	1	Clinical note	1
Maltreatment	0	0%	N/A	N/A	N/A	N/A
Victimisation	1	3.2%	Psychiatrist	1	Referral letter	1
Discrimination	0	0%	N/A	N/A	N/A	N/A
Family Mental Health Issues	0	0%	N/A	N/A	N/A	N/A
Neglect	4	12.9%	Psychiatrist	2	Clinical note	4
Bullying	9	29%	Psychiatrist	5	Clinical note	8
Death of a Sibling	1	3.2%	Peer Support Worker	1	Clinical note	1
Domestic Violence	6	19.4%	Care-coordinator	3	Clinical note	6
Parent in Prison	0	0%	N/A	N/A	N/A	N/A

Total number of times recorded is greater than 31 and the percentage of total records is greater than 100% due to some service-users’ records having more than one type of adverse experience recorded

## Discussion

This study aimed to explore how frequently adverse experiences were being recorded in the clinical records of service-users of one UK EIP service. The results showed that 64.6% of service-users’ records mentioned at least one adverse experience, with this adverse experience most commonly recorded in the service-user’s individual clinical notes by a psychiatrist. Of the 64.6% of records with one adverse experience recorded, 80.6% mentioned more than one adverse experience and 41.9% contained three or more adverse experiences. The modal number of adverse experiences recorded was two.

The results indicate that the EIP community mental health service in this study is recording adverse experiences at a similar rate to the only other study investigating this in an EIP service (Reeder et al., 2017). However, Reeder et al. (2017) only looked at childhood adverse experiences whereas the current study looked at adverse experiences in both childhood and adulthood. When the results are compared to previous literature that looked at the recording of both childhood and adulthood adverse experiences within UK CMHTs (Neill & Read, 2022), the results in this study show that adverse experiences are being recorded more frequently than in other mental health settings (64.6% in the current study compared to 13% in Neill & Read, 2022). This is particularly important when considering Neill and Read (2022) indicated that people with psychosis were less likely to have adverse experiences recorded in their clinical records. This may indicate that people with psychosis who are under the care of a CMHT may not have adverse experiences documented as frequently compared to those under the care of an EIP community mental health service.

However, previous literature indicates the prevalence rate of adverse experiences within service-users experiencing FEP to be 89% (Trauelsen et al., 2015) which is higher than the 64.6% of records found to contain at least one adverse experience in the current study. This indicates that despite the Department of Health (2008) guidelines recommending that all mental health staff routinely ask service-users about adverse experiences that this may not be happening with all service-users in this EIP service. Alternatively, it may be that service users are routinely being asked, with instances not being reported using the terms searched for in this study. The results in this study also showed that 71% of the time adverse experiences were recorded by either psychiatrists or mental health nurses and that on no occasions were adverse experiences recorded by six different professions including psychologists and CBT therapists. This may indicate that not all mental health staff within this EIP service are routinely asking about adverse experiences. However, as the profession of the clinician was only noted for the first time each adverse experience was recorded, this result may be skewed by the first point of contact within an EIP service often being with a psychiatrist or a mental health nurse.

The most common individual adverse experience search term to be recorded in this study was “trauma”, with several search terms (maltreatment, discrimination, family mental health) not being recorded in any service-user records. This may indicate that clinicians feel more comfortable enquiring about generic trauma rather than asking about specific adverse experiences, in which case further training to increase clinician’s confidence on enquiring about specific adverse experiences may be needed. Alternatively, it may be that other adverse experiences are being recorded using other terminology to those included as search terms in the study.

### Strengths and Limitations

This study was the first to look at the recording of both childhood and adulthood adverse experiences in service-user records within a UK EIP service. Therefore, it further enhances the knowledge around how adverse experiences are being recorded within service-user records of people experiencing FEP. The sample age range (19–61 years) is consistent with the age profile typically observed among individuals accessing EIP services (14–65 years) supporting clinical relevance of the sample, although the study did not undertake statistical tests to confirm representativeness of the sample.

The study is limited due to being small-scale audit with one main researcher and limited time, and methodological implications of this. Only 22 adverse experience terms were searched for, and service-user records were not read in their entirety. It is plausible that adverse experiences that had been recorded may have been missed due to deviations in terminology from the chosen search terms. Furthermore, only the first time each search term was documented, where and by whom was recorded were considered within the study. Therefore, no inferences can be made regarding further reporting of adverse experiences. The study also only looked at a relatively small sample within one EIP community mental health service, therefore results cannot be generalised to other services. A further limitation is that due to service limitations only 49 of a potential 98 records were included in the analysis, meaning the results may not be representative of the whole service. Future research with larger scale and scope should endeavour to include all eligible records in their analysis to ensure conclusions that can be definitively attributed to the whole service provision.

Finally, the study only looked at the service-users’ records and did not directly ask the service-users or clinicians regarding whether adverse experiences were directly asked about. Thus, it cannot be ascertained if the number of adverse experiences recorded are a true reflection of the adverse experiences actually experienced by the service-users or asked about by clinicians as per practice guidelines.

### Clinical and Research Implications

Clinically, the study suggests the reporting of adverse experiences in client notes and documentation is higher in the current service than has been reported in other services (Chandon et al., 2020; Neill & Read et al., 2022; (Read et al., 2018a, b)). Further research is needed to see if this is the case across the UK within other EIP community mental health services, particularly due to the known high prevalence rate of adverse experiences in people experiencing FEP (Aas et al., 2016; Trauelsen et al., 2015). Reporting of adverse experiences in this way will help to ensure service-users receive the most appropriate trauma-informed care while under the care of an EIP community mental health service. This is important as trauma-informed care reduces the risk of retraumatising the service-user, facilitates better relationships with clinicians and increases the likelihood of accessing mental health services again (Piotrowski, 2020). Trauma-informed care can improve treatment outcomes for service-users who have experienced adverse life experiences (Reeves, 2015).

Further research could investigate what variables, such as clinician confidence, may lead to variance in reporting adverse experiences across services. Further research could also consider if the use of routine screening tools during initial assessments, like the TALE checklist (Carr et al., 2018) and ACE questionnaire (Felitti et al., 1998) increases the chance of adverse experiences being enquired about and accurately documented. It may also be beneficial for future research to consider using broader search terms or to read service-users’ records in their entirety to further understand the prevalence rate of adverse experiences in this population.

Although this study looked at 22 different adverse experience search terms, it was difficult to compare the findings for individual search terms to previous literature due to many studies only looking at childhood adverse experiences (Bebbington et al., 2004; Reeder et al., 2017; Varese et al., 2012). More research is needed to explore the prevalence rate of lifetime adverse experiences on the risk of experiencing psychosis so that this can be used to aid the training of mental health staff in asking about and the accurate documentation of adverse experiences in service-user records. Developing training to support healthcare professionals to inquire about adverse experiences may increase the frequency in which adverse experiences are asked about (Lotzin et al., 2018) and reduce potential barriers to asking about adverse experiences which in turn may further improve the detection rate of these experiences (Lotzin et al., 2019).

Finally, it would be beneficial for future research to ask service-users of EIP community mental health services directly about adverse experiences, and to compare the prevalence rate to what has been documented in service-user records. This method would allow for inferences to be made regarding whether practice guidance is being followed (with regards to asking), and whether documentation in records is an accurate representation of whether service users are being asked about this or not.

## Conclusion

This study found that 64.6% of service-user records in a UK EIP community mental health service documented at least one lifetime adverse experience, indicating this service may routinely ask more frequently than other mental health settings about adverse experiences. However, the rate documented is lower than the prevalence rate of adverse experiences reported in the literature for service-users with FEP. Further research across a wider number of EIP community mental health services is needed to see if this result is generalisable and if so, whether documentation accurately reflects the extent to which service users are being asked about this in clinical practice.
